# Influence of HLA-DRB1 and HLA-DQB1 Alleles on IgG Antibody Response to the *P. vivax* MSP-1, MSP-3α and MSP-9 in Individuals from Brazilian Endemic Area

**DOI:** 10.1371/journal.pone.0036419

**Published:** 2012-05-23

**Authors:** Josué C. Lima-Junior, Rodrigo N. Rodrigues-da-Silva, Dalma M. Banic, Jianlin Jiang, Balwan Singh, Gustavo M. Fabrício-Silva, Luís C. S. Porto, Esmeralda V. S. Meyer, Alberto Moreno, Maurício M. Rodrigues, John W. Barnwell, Mary R. Galinski, Joseli de Oliveira-Ferreira

**Affiliations:** 1 Laboratory of Immunoparasitology, Instituto Oswaldo Cruz, Fiocruz, Rio de Janeiro, Brazil; 2 Center for Technological Development in Health (CDTS), Oswaldo Cruz Foundation (Fiocruz), Rio de Janeiro, Brazil; 3 Laboratório de Simulídeos e Oncocercose, Instituto Oswaldo Cruz, Fiocruz, Rio de Janeiro, Brazil; 4 Emory Vaccine Center, Emory University, Atlanta, Georgia, United States of America; 5 Histocompatibility and Cryopreservation Laboratory, Rio de Janeiro State University, Rio de Janeiro, Brazil; 6 Centro de Terapia Celular e Molecular (CTCMol), Universidade Federal de São Paulo, Escola Paulista de Medicina, São Paulo, Brazil; 7 Division of Infectious Diseases, Emory University School of Medicine, Atlanta, Georgia, United States of America; 8 Division of Parasitic Diseases, CDC/National Center for Infectious Diseases, Atlanta, Georgia, United States of America; Universidade Federal de Minas Gerais, Brazil

## Abstract

**Background:**

The antibody response generated during malaria infections is of particular interest, since the production of specific IgG antibodies is required for acquisition of clinical immunity. However, variations in antibody responses could result from genetic polymorphism of the HLA class II genes. Given the increasing focus on the development of subunit vaccines, studies of the influence of class II alleles on the immune response in ethnically diverse populations is important, prior to the implementation of vaccine trials.

**Methods and Findings:**

In this study, we evaluated the influence of HLA-DRB1* and -DQB1* allelic groups on the naturally acquired humoral response from Brazilian Amazon individuals (n = 276) against *P. vivax* Merozoite Surface Protein-1 (MSP-1), MSP-3α and MSP-9 recombinant proteins. Our results provide information concerning these three *P. vivax* antigens, relevant for their role as immunogenic surface proteins and vaccine candidates. Firstly, the studied population was heterogeneous presenting 13 HLA-DRB1* and 5 DQB1* allelic groups with a higher frequency of HLA-DRB1*04 and HLA-DQB1*03. The proteins studied were broadly immunogenic in a naturally exposed population with high frequency of IgG antibodies against PvMSP1-19 (86.7%), PvMSP-3 (77%) and PvMSP-9 (76%). Moreover, HLA-DRB1*04 and HLA-DQB1*03 alleles were associated with a higher frequency of IgG immune responses against five out of nine antigens tested, while HLA-DRB1*01 was associated with a high frequency of non-responders to repetitive regions of PvMSP-9, and the DRB1*16 allelic group with the low frequency of responders to PvMSP3 full length recombinant protein.

**Conclusions:**

HLA-DRB1*04 alleles were associated with high frequency of antibody responses to five out of nine recombinant proteins tested in Rondonia State, Brazil. These features could increase the success rate of future clinical trials based on these vaccine candidates.

## Introduction

Malaria is one of the most prevalent parasitic diseases in tropical and subtropical countries. About 500 million new cases are reported annually, and it is estimated that around 1–2 million of these cases are fatal [1]. *Plasmodium vivax* is the most widespread malaria species affecting mainly Asian, South and Central American countries, and the second leading cause of malaria, responsible for 132–391 million infections per year [2].

Vaccination is considered one of the most promising strategies for controlling this disease [3], however *Plasmodium* species have a complex life cycle involving a mosquito vector and a vertebrate host [4]. During asexual stage development merozoite surface proteins and proteins released from the apical organelles (rhoptries, micronemes, and dense granules) are responsible for the cascade of events involved in the parasite invasion of red blood cells (RBCs) [5]. In this context, the family of merozoite surface proteins (MSPs) seems to be important for the first contact between merozoites and RBCs and they have therefore become important targets for vaccine development [6], [7], [8], [9]. In *P. vivax*, the merozoite surface proteins (MSPs) PvMSP-1[10], the PvMSP-3 family [11], [12] and PvMSP-9 [13], [14] are currently recognized as vaccine candidates. The potential of these proteins as vaccine candidates is based on their recognition by antibodies from individuals naturally exposed to *P. vivax* [15], [16], [17], [18], their immunogenic properties in animal models [19], [20], [21], and evidence of the induction of parasite growth-inhibitory specific antibodies [11], [13], [22].

The importance of antibody responses during malaria infection has long been observed, and the production of specific IgG antibodies is required for the acquisition of clinical immunity. In our previous studies we assessed naturally acquired humoral immune responses against PvMSP-3α and PvMSP-9. Our first set of data on antibody responses show that these proteins are targets of the immune response in individuals naturally exposed to *P. vivax* malaria transmission [15], [16], [17], [18]. However, the population of non-responders ranged from 21% for PvMSP-3α to 58.7% for PvMSP-9.

We hypothesized that variations in antibody response could be determined by genetic polymorphism of the Human Leukocyte Antigens (HLA) class II genes and sought to relate the antibody response to specific HLA alleles and haplotypes. There is a significant body of evidence that the genes affecting the immune response can influence the outcome of malaria infection and the capacity to mount a humoral immune response [23], [24]. HLA class II genes were originally called immune response genes, since their alleles are known to influence the antibody response. Exogenous as well as endogenous peptides are presented in the context of HLA class II molecules for recognition by CD4+ T lymphocytes. CD4+ T cell subsets produce cytokines that provide help to B cells for antibody production [25]. In humans, MHC class II molecules are encoded by three different loci, HLA-DR, -DQ, and -DP, which display 70% similarity to each other. Polymorphism is a notable feature of MHC class II genes. For HLA-DR, most variability comes from DRB, with >700 known alleles at the population level, whereas there are only three DRA variants. In contrast, both chains of HLA-DQ and -DP are polymorphic. However for HLA-DP, only a few alleles are prevalent [26].

The role played by the HLA system on the immune response to malaria antigens has been extensively investigated given the relevance of HLA-restricted immune responses for the development of subunit vaccines [27], [28], [29]. Although defined host–parasite interactions at the level of antigen processing and presentation might affect the development of specific immune responses, the available evidence indicates that HLA loci are an important genetic determinant of the immune reactivity to malaria. Several authors have reported an association between HLA class II alleles and the acquisition of antibodies to B-cell epitopes in the *P. falciparum* ring-infected erythrocyte surface antigen (RESA), the P126 antigen, the glutamate-rich protein (GLURP), the subunit vaccine candidate SPf66, and to the repeat region of the *P. vivax* circumsporozoite protein (CSP) [30], [31], [32], [33], [34], [35], [36], [37], [38].

There is an increasing focus on the development of subunit malaria vaccines, and studies of the influence of class II alleles on the immune response in ethnically diverse populations is important before implementation of vaccine trials. This is particularly relevant for *P. vivax*, which is the most widespread malaria parasite, affecting populations with a high diversity of genetic backgrounds. Therefore, in this study, we evaluated the influence of HLA-DRB1* and -DQB1* allelic products on the frequency of naturally acquired antibodies to *P. vivax* MSP-1, MSP-3α and MSP-9 recombinant proteins.

## Results

### Epidemiological Characteristics of Studied Population

Our epidemiological survey, summarized in Table 1, shows that all individuals studied were exposed to malaria infections throughout the year. A significant proportion of studied individuals reported a prior experience with *P. vivax* or *P. falciparum* malaria when compared with individuals that could not recall infections in the past and mentioned that they never had malaria even though they were born in the endemic area (*p*<0.0001). Among donors with a previous malaria infection(s), years of residence in the endemic area correlated positively with the past months since last malaria episode (Spearman’s r = 0.2411, P<0.0001, n = 245). At the time of blood collection 34 individuals were infected, 25 with *P. vivax* and 9 with *P. falciparum* (thus, 74.6% of the infected individuals had *P. vivax* and 26.4% had *P. falciparum*), consistent with the current local case distribution data for these two species reported by the Brazilian Ministry of Health [39].

**Table 1:** Summary of the epidemiological characteristics of studied individuals enrolled in this work.

Epidemiological characteristics	
Gender	
Male (n)	152
Female (n)	124
TOTAL (n)	276
Age (Mean ± SD)	36.1+16.4
Time of residence in malaria endemic area (Mean ± SD)	28.5+17.0
Number of past malaria infections (Mean ± SD)	7.0+9.6
Number of malaria infections in the last 6 months(Mean ± SD)	0.5+1.1
Past months since the last malaria infection (Mean ± SD)	41.0+50.1
Hospitalization in malaria past infections^b^ (n/%)	56/19.8%
Use of prophylactic measures^e^	131/46.5%
Previous malaria species contracted^c^	
Negative (n)	31/11.3%
* P. vivax* (n)	56/20,2%
* P.falciparum* (n)	34/12.3%
Both species (n)	**155/56.2%**

a Differences in gender proportions were not statistically significant (χ^2^ = 1.7281; P = 0.3447).

b We could not determine if clinical criteria were compatible with severe malaria,

c 11 (3.2%) individuals did not remember the previous malaria parasite species contracted.

d Bold typeface indicates that the prevalence was significantly higher when compared with all other observations (P<0.0001).

e Were considered as prophylactic measure; i.e., the use of residual insecticide, bednets or insect repellent.

### HLA-DRB1 and HLA-DQB1 Allele Frequencies of Studied Individuals

**Figure 1:**
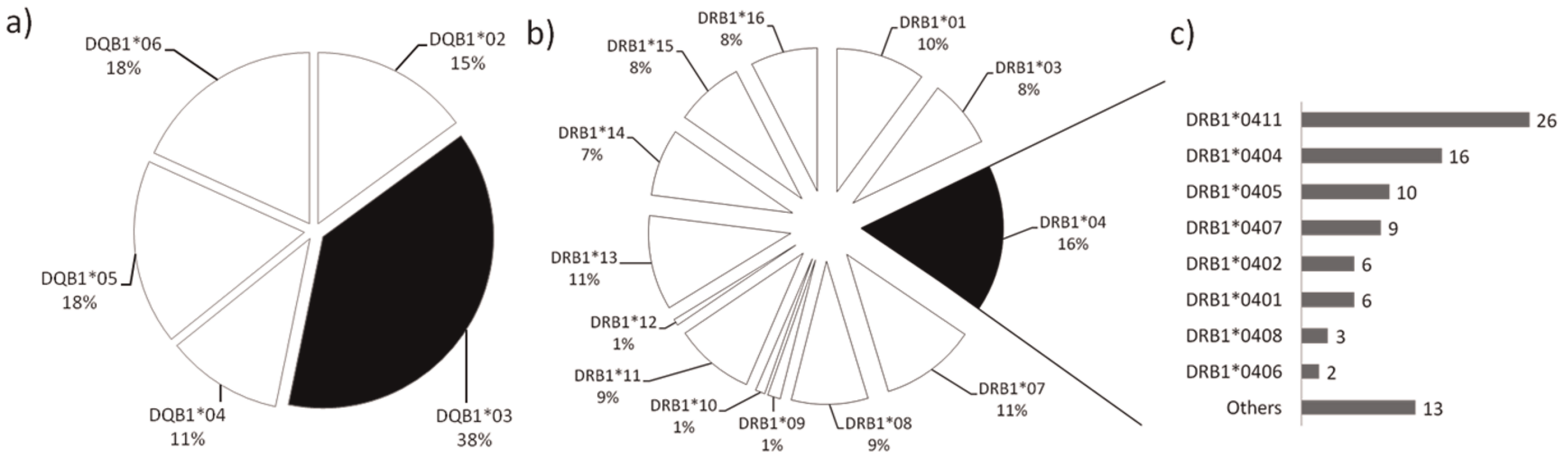
HLA allele frequencies in individuals naturally exposed to malaria from the Rondonia state. HLA-DQB1* (a) and HLA-DRB1* (b) allele frequencies (%) in 276 individuals naturally exposed to malaria from the Rondonia state enrolled in our study. HLA-DQB1*03 and HLA-DRB1*04 present significantly higher frequencies when compared with all other groups observed (P<0,05 and P<0,01 respectively). Others: DRB1*09, DRB1*10, DRB1*12. The high resolution allele frequency distribution among the 91 HLA-DRB1*04 carriers from our studied area (c) also show a large variety of alleles, with a marked predominance of HLA-DRB1*04∶11 (28%) over the others (P<0.05).

HLA-DRB1* and HLA-DQB1* typing was performed on DNA samples of 276 individuals included in the cohort. Both the number of positive individuals for the HLA-DQB1* and HLA-DRB1* alleles and the frequency of each allele are summarized in Figures 1a and 1b. We found 13 HLA-DRB1* and 5 HLA-DQB1* allelic groups. There were two predominant HLA allelic groups in our studied population, HLA-DRB1*04 (16%) of all HLA-DR genotypes, and HLA-DQB1*03 (38%) of all HLA-DQ genotypes. The HLA-DRB1*09, HLA-DRB1*10 and HLA-DRB1*12 were less frequent in HLA-DRB1* and HLA-DQB1*04 in HLA-DQB1*. As observed in Figure 1c, HLA-DRB1*04 carrier individuals also show a large variety of alleles, with a marked predominance of HLA-DRB1*0411 (28%) over the others (P<0.05). 

### Frequency of IgG Response to PvMSP-1, PvMSP-3α and PvMSP-9 and Associations with HLA-DRB1* and HLA-DQB1* Alleles

**Figure 2:**
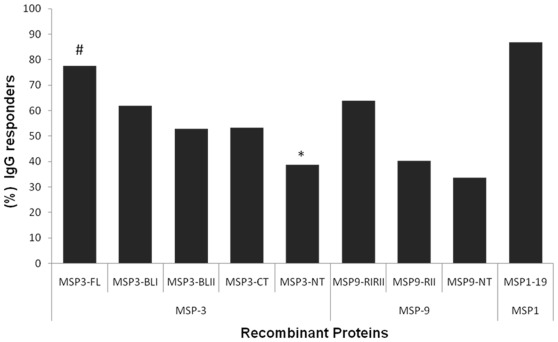
Frequency of IgG responders to PvMSP-1, PvMSP-3 and PvMSP-9. Frequency of IgG responders to five recombinant proteins representing different regions of PvMSP-3α, three recombinant proteins representing PvMSP-9 and PvMSP-1_19_ in the studied population. Chi squared test for proportions analyses were performed to determine statistical differences. # The frequency of IgG responders to PvMSP3-FL was significantly higher when compared with all others PvMSP-3α recombinants (P<0.05) * The frequency to PvMSP3-NT was the lowest when compared with all others (P<0.01), and the frequencies to PvMSP9-RIRII were higher (P<0,01) when compared with other PvMSP-9 recombinants.

The prevalence of naturally acquired antibodies specific to the recombinant proteins was determined in plasma of 276 studied individuals (Figure 2). The frequency of responders show that IgG antibody reactivity to PvMSP1_19_ was 86.7% and the frequency of positive individuals for at least one of the recombinant proteins representing PvMSP-3α and PvMSP-9 sequences was 77% and 76%, respectively. The recombinant protein representing PvMSP1_19_ was the most recognized of the proteins tested. A substantial variation in terms of recognition was observed for the different regions of PvMSP-3α. The two blocks of repeats (MSP3_RI_, 62% and MSP3_RII_, 53%) and the C-terminal regions (MSP3_CT_, 53%) were significantly more recognized (P<0.01) than the N-terminal region (MSP3_NT_, 39%). Similarly, the recombinant representing the two blocks of repeats in MSP9_RIRII_ (63.7%) were significantly higher when compared to the recombinant representing only the second block of repeats (MSP9_RII_, 40.2%; χ^2^ = 10.3; P<0.0013) and the N terminal region (PvMSP9_NT,_ 33.7%; χ^2^ = 15.2; P<0.0001). Combining data from all recombinant proteins a proportion of the population appears to be non-responders for PvMSP-1 (13%), PvMSP-3α (22.4%) and PvMSP-9 (28.7%) and five individuals were non-responders for all recombinant proteins.

**Figure 3:**
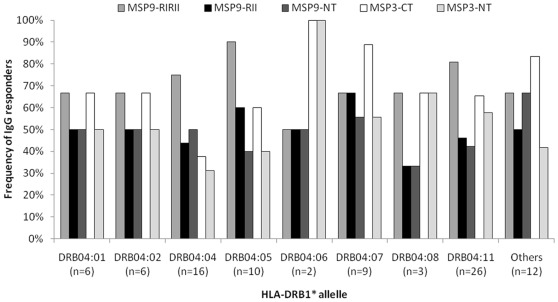
Frequency (%) of responders to PvMSP9 and PvMSP-3 recombinant proteins by HLA-DRB1*04 alleles. Frequency (%) of responders to PvMSP9 and PvMSP-3 recombinant proteins by HLA-DRB1*04 alleles. The frequencies of responders were not associated to a particular HLA-DRB1*04 allelic group by the bipartition χ^2^ test (p>0.05). * “Others" category groups individuals with HLA-DRB1*04 less frequent alleles.

The influence of HLA-DRB1* and HLA-DQB1* alleles and haplotypes on the naturally acquired IgG response to the recombinant proteins representing PvMSP-1, PvMSP-3α and PvMSP-9 was also evaluated (Tables 2 and 3). Although 13% of the population did not present detectable titers of antibodies to PvMSP-1 (MSP1_19_), genetic restriction to this antigen does not seem to occur, since no association was observed between the HLA DRB1* or DQB1 alleles and the antibody response to MSP1_19._ However for the PvMSP-3α and PvMSP-9 recombinants proteins, the IgG responders were associated with the presence of the HLA-DRB1*04 allelic group. The associations were found in IgG responders to the recombinants proteins representing the C-terminal and N-terminal regions of PvMSP-3α (MSP3_CT_, P<0.025; MSP3_NT_ P<0.025) and the two blocks of repeats and the N-terminal region of PvMSP-9 (MSP9_RIRII_, P<0.05; MSP9_RII_, P<0.05; MSP9_NT_, P<0.025). Among the individuals presenting the HLA-DRB1*04 allelic group the frequency of responders to all recombinant proteins was higher in the HLA-DRB1*0411 allele carriers. However, the association was not statistically significant with any specific HLA-DRB1*04 allele (figure 3).

**Table 2:** Frequency (F) and number (n) of IgG responders and non-responders to the PvMSP-3α recombinant proteins tested by HLA-DRB1* and HLA-DQB1*allelic groups from malaria naturally exposed individuals.

HLA	MSP3FL		MSP3BLI		MSP3BLII		MSP3CT		MSP3NT	
	Responder	Non-responder	Responder	Non-responder	Responder	Non-responder	Responder	Non-responder	Responder	Non-responder
	*f (n)*	*f (n)*	*f (n)*	*f (n)*	*f (n)*	*f (n)*	*f (n)*	*f (n)*	*f (n)*	*f (n)*
***HLA-DRB1*** *										
*DRB01*	0.105 (45)	0.089 (11)	0.114 (39)	0.081 (17)	0.106 (31)	0.096 (25)	0.085 (25)	0.120 (31)	0.089 (19)	0.109 (37)
*DRB03*	0.079 (34)	0.073 (9)	0.076 (26)	0.081 (17)	0.086 (25)	0.069 (18)	0.078 (23)	0.078 (20)	0.098 (21)	0.065 (22)
*DRB04*	0.164 (70)	0.169 (21)	0.178 (61)	0.143 (30)	0.168 (49)	0.162 (42)	**0.201 (59)**	**0.124 (32)**	**0.206 (44)**	**0.139 (47)**
*DRB07*	0.110 (47)	0.105 (13)	0.105 (36)	0.114 (24)	0.103 (30)	0.115 (30)	0.116 (34)	0.101 (26)	0.084 (18)	0.124 (42)
*DRB08*	0.091 (39)	0.073 (9)	0.082 (28)	0.095 (20)	0.089 (26)	0.085 (22)	0.075 (22)	0.101 (26)	0.103 (22)	0.077 (26)
*DRB09*	0.019 (8)	0.0 (0)	0.012 (4)	0.019 (4)	0.014 (4)	0.015 (4)	0.014 (4)	0.016 (4)	0.019 (4)	0.012 (4)
*DRB10*	0.009 (4)	0.016 (2)	0.012 (4)	0.010 (2)	0.014 (4)	0.080 (2)	0.010 (3)	0.012 (3)	0.0 (0)	0.018 (6)
*DRB11*	0.098 (42)	0.065 (8)	0.091 (31)	0.090 (19)	0.089 (26)	0.092 (24)	0.095 (28)	0.085 (22)	0.089 (19)	0.092 (31)
*DRB12*	0.009 (4)	0.0 (0)	0.009 (3)	0.003 (1)	0.010 (3)	0.004 (1)	0.010 3)	0.004 (1)	0.014 (3)	0.003 (1)
*DRB13*	0.103 (44)	0.121 (15)	0.114 (39)	0.095 (20)	0.103 (30)	0.112 (29)	0.102 (30)	0.112 (29)	0.098 (21)	0.112 (38)
*DRB14*	0.072 (31)	0.089 (11)	0.067 (23)	0.090 (19)	0.075 (22)	0.077 (20)	0.085 (25)	0.066 (17)	0.056 (12)	0.089 (30)
*DRB15*	0.077 (33)	0.081 (10)	0.076 (26)	0.081 (17)	0.082 (24)	0.073 (19)	0.061 (18)	0.097 (25)	0.079 (17)	0.077 (26)
*DRB16*	**0.063 (27)**	**0.121 (15)**	0.064 (22)	0.095 (20)	0.062 (18)	0.092 (24)	0.068 (20)	0.085 (22)	0.065 (14)	0.083 (28)
***HLA-DQB1*** *										
*DQB02*	0.153 (66)	0.137 (17)	0.145 (50)	0.157 (33)	0.140 (41)	0.160 (42)	0.156 (46)	0.142 (37)	0.140 (30)	0.156 (53)
*DQB03*	0.384 (165)	0.379 (47)	0.384 (132)	0.381 (80)	0.370 (108)	0.397 (104)	**0.435 (128)**	**0.323 (84)**	0.416 (89)	0.362 (123)
*DQB04*	0.112 (48)	0.105 (13)	0.305 (36)	0.119 (25)	0.116 (34)	0.103 (27)	0.099 (29)	0.123 (32)	0.117 (25)	0.106 (36)
*DQB05*	0.172 (74)	0.185 (23)	0.186 (64)	0.157 (33)	0.185 (54)	0.164 (43)	0.163 (48)	0.188 (49)	0.145 (31)	0.194 (66)
*DQB06*	0.179 (75)	0.194 (24)	0.180 (60)	0.186 (39)	0.188 (53)	0.176 (46)	**0.146 (41)**	**0.223 (58)**	0.182 (37)	0.182 (62)

Bold typeface indicates the frequency of IgG responses were associated to the particular HLA-DRB1* or HLA-DQB1* allelic group by the bipartition χ^2^ test (P<0.05).

Each studied individual contributed two HLA allele observations to this descriptive analysis.

**Table 3:** Frequency (F) and number (n) of IgG responders and non-responders to the PvMSP-9 and PvMSP-1 recombinant proteins tested by HLA-DRB1* and HLA-DQB1*allelic groups from malaria naturally exposed individuals.

HLA	MSP9-RIRII		MSP9-RII		MSP9-NT		MSP1	
	Responder	Non-responder	Responder	Non-responder	Responder	Non-responder	Responder	Non-responder
	*f (n)*	*f (n)*	*f (n)*	*f (n)*	*f (n)*	*f (n)*	*f (n)*	*f (n)*
HLA-DRB1*								
*DRB01*	**0.065 (23)**	**0.160 (32)**	**0.063 (14)**	**0.124 (41)**	0.086 (16)	0.107 (39)	0.090 (43)	0.151(11)
*DRB03*	0.074 (26)	0.085 (17)	0.086 (19)	0.073 (24)	0.075 (14)	0.079 (29)	0.079(38)	0.068 (5)
*DRB04*	**0.190 (67)**	**0.120 (24)**	**0.203 (45)**	**0.139 (46)**	**0.231 (43)**	**0.132 (48)**	0.159 (76)	0.205 (15)
*DRB07*	0.097 (34)	0.120 (24)	0.131 (29)	0.088 (29)	0.108 (20)	0.104 (38)	0.106 (51)	0.123 (9)
*DRB08*	0.097 (34)	0.065 (13)	0.095 (21)	0.079 (26)	0.081 (15)	0.088 (32)	0.088 (42)	0.082 (6)
*DRB09*	0.011 (4)	0.020 (4)	0.014 (3)	0.015 (5)	0.022 (4)	0.011 (4)	0.015 (7)	0.041 (3)
*DRB10*	0.009 (3)	0.015 (3)	0.0 (0)	0.018 (6)	0.005 (1)	0.014 (5)	0.013 (6)	0.0(0)
*DRB11*	0.116 (41)	0.060 (12)	0.077 (17)	0.109 (36)	0.091 (17)	0.099 (36)	0.096 (46)	0.055 (4)
*DRB12*	0.009 (3)	0.005 (1)	0.009 (2)	0.006 (2)	0.0 (0)	0.008 (3)	0.006 (3)	0.014 (1)
*DRB13*	0.102 (36)	0.110 (22)	0.104 (23)	0.106 (35)	0.086 (16)	0.115 (42)	0.111 (53)	0.082 (6)
*DRB14*	0.065 (23)	0.100 (20)	0.072 (16)	0.082 (27)	0.075 (14)	0.079 (29)	0.084 (40)	0.027 (2)
*DRB15*	0.082 (29)	0.075 (15)	0.072 (16)	0.085 (28)	0.075 (14)	0.082 (30)	0.075 (36)	0.096 (7)
*DRB16*	0.082 (29)	0.065 (13)	0.077 (17)	0.076 (25)	0.065 (12)	0.082(30)	0.079 (38)	0.055 (4)
HLA-DQB1*								
*DQB02*	0.134 (47)	0.165 (33)	0.179 (40)	0.121 (40)	0.124 (23)	0.155 (57)	0.143 (68)	0.154 (12)
*DQB03*	**0.432 (152)**	**0.315 (63)**	0.424 (95)	0.364 (120)	**0.473 (88)**	**0.345 (127)**	0.399(190)	0.321 (25)
*DQB04*	0.131 (46)	0.080 (16)	0.129 (29)	0.100 (33)	0.129 (24)	0.103 (38)	0.109 (52)	0.128 (10)
*DQB05*	0.139 (49)	0.235 (47)	0.125 (28)	0.206 (68)	0.140 (26)	0.190 (70)	0.168 (80)	0.205 (16)
*DQB06*	0.165 (58)	0.205 (41)	0.143 (30)	0.209 (69)	0.134 (23)	0.207 (76)	0.181 (86)	0.192 (15)

Bold typeface indicates the frequency of IgG responses were associated to the particular HLA-DRB1* or HLA-DQB1* allelic group by the bipartition χ^2^ test (P<0.05).

Each studied individual contributed two HLA allele observations to this descriptive analysis.

The high frequency of non-responders to the recombinant proteins representing the blocks of repeats of PvMSP-9 (MSP9_RIRII_ and MSP9_RII_) is associated with the presence of the HLA-DRB1*01 allelic group (P<0.001 and P<0.005 respectively) and the recombinant protein representing the full length of PvMSP-3α with the presence of the HLA-DRB1*16 allelic group (MSP3_FL_) (P<0.05). Interestingly, this association was not detected when we evaluated the response to the recombinant proteins representing the different regions of PvMSP3.

In the evaluation of the HLA-DQB1* allelic groups we observed an association between HLA-DQB1*03 and responders to the recombinant protein representing the repeats and the N-terminal region of PvMSP-9 (MSP9_RIRII_ P<0.05; MSP9_NT_ P<0.05) and the C-terminal region of PvMSP-3α (MSP3_CT_, P<0.01). In contrast, a negative association was observed between HLA-DQB1*06 and responders to the C-terminus of PvMSP-3α (MSP3_CT_, P<0.025). All HLA associations with specific antibodies against PvMSP-1, PvMSP-3 and PvMSP-9 are summarized in table 4.

**Table 4:** Summary of associations between HLA-DRB1* allelic groups and the frequency of responders to PvMSP-1, PvMSP-3α and PvMSP-9 calculated by the bi-partition X^2^ test.

Protein	HLA	Associations	P
***PvMSP3-NT***	DRB1*04	(+)	<0.025
***PvMSP3-CT***	DRB1*04	(+)	<0.025
	DQB1*03	(+)	<0.01
	DQB1*06	(−)	<0.025
***PvMSP3-FL***	DRB1*16	(−)	<0.05
***PvMSP9-RIRII***	DRB1*01	(−)	<0.001
	DRB1*04	(+)	<0.05
***PvMSP9-RII***	DRB1*01	(−)	<0.005
	DRB1*04	(+)	<0.05
***PvMSP9-NT***	DRB1*04	(+)	<0.025

### Effect of HLA and TREA, PMI and TLI on Antibody Levels to PvMSP-1, PvMSP-3α and PvMSP-9

The effects of different HLA-DRB1 and HLA-DQB1 allelic groups and time of residence (years) in malaria endemic areas on the level of antibodies to PvMSP-1, PvMSP-3α and PvMSP-9, were also analyzed. The DRB1 and DQB1 alleles that are observed frequently in the population were included in these analyses. Multivariate analyses were performed by using time (years) of residence in endemic area (TREA), time (months) since the last malaria episode (TLI) and past malaria infections (PMI) and DRB1 or DQB1 alleles as independent variables and the reactivity indexes of IgG against all studied recombinant proteins as dependent variables. A significant association was observed only between HLA-DR+TREA and the reactivity index of IgG against PvMSP9-RIRII (F = 1.274, P = 0.0333) and PvMSP9-RII (F = 1.259, P = 0.0401). Interestingly, to all PvMSP-9 antigens, the observed power of association of each independent variable separately was greater in TREA (0.097 to 1.000) than in the HLA allelic groups (0.345 to 0.643). Therefore, the time of exposure is more closely related to the PvMSP-9 antibody levels. In addition the observed power of association was similar between both variables. No significant associations, positive or negative, were observed with HLA-DR+TREA, HLA-DR+PMI, HLA-DR+TLI and antibody levels to PvMSP-3α and PvMSP-1. No significant associations were observed for any HLA-DQ+TREA, HLA-DQ+PMI, HLA-DQ+TLI and PvMSP-1, PvMSP-3α and PvMSP-9 antibody levels.

### HLA-DRB1*04 Carriers Presented Higher Levels of Antibodies to PvMSP-9 but not to PvMSP-1 and PvMSP-3α

Since the DRB1*04 allelic group was associated with responders, we also evaluated if the levels of antibodies, represented by the reactivity indexes to the recombinant proteins in the ELISA test, had the influence of these alleles. Comparing the antibody response in carriers and non-carriers of the HLA-DRB1*04 alleles, the levels were significantly higher in HLA-DRB1*04 carriers for the recombinant proteins representing PvMSP-9: MSP9_RIRII_ (carriers Mean ± SD = 3.23±0.60 vs non-carriers Mean ± SD = 1.95±0.47; df = 254, t = 2.934, P = 0.0037), MSP9_RII_ (carriers Mean ± SD = 2.49±0.41 vs non-carriers Mean ± SD = 1.41±0.23; df = 188, t = 2.923, P = 0.0045) and PvMSP9_NT_ (carriers Mean ± SD = 2.01±0.29 vs non-carriers Mean ± SD = 1.37±0.19; df = 124, t = 5.975, P<0.0001). However we did not find any difference in the levels of antibodies between these groups for the PvMSP-1 and PvMSP-3α responders. The antibody levels in individuals homozygous for the HLA-DRB1*04 allelic group did not differ from individuals that carry only one DRB1*04 allelic group (heterozygous) independent of the recombinant protein recognized. Finally, no relationship between a particular HLA haplotype and the frequency of antibody response was observed.

## Discussion

Many factors can contribute to the heterogeneity of the immune response to vaccines, including polymorphism of immune response genes. Understanding the genetic restriction that influences the generation of protective immune responses to *Plasmodium* target proteins is important to develop novel vaccines and improve our knowledge about current vaccine candidates. Not only biochemical and structural studies of class II MHC molecules, but also studies in specific immune responses to recombinant proteins or synthetic peptides, provide a conceptual foundation for the rational design of subunit vaccines.

In our study, differences in the frequency of antibodies to three merozoite surface proteins were defined in a population similarly exposed to malaria infections. The proportion of non-responders varied from 13% for PvMSP-1 to 28.7% for PvMSP-9. Five individuals were non-responders for all recombinant proteins tested here. Differences in malaria morbidity and antibody responses to several *Plasmodium* antigens suggest genetic regulation of the immune responses [23], [24]. We hypothesized that differences in the malaria antibody response to merozoite antigens could be explained by the genetic polymorphism of the HLA Class II alleles. However, repeated sequences, common in *Plasmodium* proteins, are immunodominant [36], [40], [41]. Similarly, sequence variation in allelic forms may prevent the development of cross-reactive antibodies [42], [43].

The studied population was heterogeneous presenting 13 HLA-DRB1* and 5 DQB1* allelic groups. As expected, the Brazilian populations have features of a tri-hybrid admixed population with contribution from Caucasian, African, and native Amerindian ancestors, in which the phenotypic characteristics of each original population have been highly mixed. However, we also observed a high frequency of HLA-DRB1*04 and HLA-DQB1*03, suggesting that in this population the Amerindian HLA genotype is conserved [44]. The enrollment of populations with high HLA polymorphism but with a related degree of conservation was ideal to observe the association of HLA allelic groups and the immune response to *P. vivax* antigens.

The analysis of the presence of HLA-DRB1* and HLA-DQB1* allelic groups and the antibody response to PvMSP1_19_ did not show any positive or negative association. Previous studies with *P. falciparum* MSP1_19_ also showed no association between the levels of antibodies to one MSP1_19_ variant and the HLA-DRB1*, DQB1* alleles or HLA-DR/DQ haplotype [30]. Therefore, our findings indicate that this region of PvMSP-1 is highly immunogenic in naturally exposed populations and the acquired immune response observed is not restricted to a particular HLA genotype [45]. Nevertheless, we showed HLA associations with the IgG immune response to the recombinant proteins representing different regions of PvMSP-3α and PvMSP-9. A high frequency of responders to PvMSP3_CT_ and PvMSP3_NT_ were defined in HLA-DRB1*04 carriers and to PvMSP3_CT_ in HLA-DQB1*03 carriers. In addition, HLA-DRB1*04 and DQB1*03 were also positively associated with the IgG response against the N-terminal and repetitive regions of PvMSP-9.

An association with specific IgG antibodies and HLA alleles has been reported for some *P. falciparum* vaccine candidates. In malaria patients, HLA-DRB1*07 alleles were correlated with improved antibody responses against both domains of the chimeric protein PfCP-2.9 (*P. falciparum* apical membrane antigen-1, PfAMA-1, and PfMSP-1_19_) but HLA-DRB1*08 had the contrary effect on PfAMA-1 and PfMSP-1_19_ [46]. A study in Cameroon Republic indicated that individuals with HLA-DRB1*1201 alleles had higher antibody responses to the recombinant PfAMA-1 variant, but there was no association between HLA-II alleles and PfMSP-1_19_ antibody levels [30]. In contrast, there are few studies reporting an HLA influence in IgG immune responses to *P. vivax* proteins, however associations were observed between antibody responses to the CSP repeats of VK247 and the presence of HLA-DRB1*16 and HLA-DRB1*07 and the lack of antibody responses to the CSP repeats of VK210 [29].

In this study, the highest response was associated with HLA-DRB1*04, the most frequent alleles found in the studied population and, coincidently, also the most frequent allele in native individuals from the Amazon [47]. However, this population had more time of exposure to malaria infections than the migrants from non-endemic areas. We also observed that the time of residence in malaria endemic areas was directly correlated with the IgG specific immune response to PvMSP-9 but not with PvMSP-1 and PvMSP-3α. Interestingly, the multivariate analysis allowed us to detect the influence of both, HLA and time of residence in a malaria endemic area (HLA+TREA) on levels of specific antibodies to PvMSP-3α and PvMSP-9. The lack of association in the interaction of HLA*TREA with the antibody levels to PvMSP-3α in our study was expected, since our previous studies demonstrated that naturally acquired antibodies raised against PvMSP-3α are not directly associated with the time of exposure in Brazilian endemic areas. Therefore the association observed was mainly with HLA-DRB1*04 and HLA-DQB1*03, suggesting that these alleles in fact could be predictors of higher antibody levels to PvMSP-3_CT_ and PvMSP-3_NT_. Conversely, we observed an association of these interactions (TREA*HLA) and specific IgG antibodies against the repetitive regions of PvMSP-9, suggesting that time of residence in the endemic areas investigated influences the antibody response rather than the HLA-DRB1* alleles.

Although the above association exists, the extensive polymorphism of HLA alleles is generally accepted to have evolved as a result of selective pressure caused by pathogens with certain HLA types [48]. If the above associations between HLA and antibodies to PvMSP-3α and PvMSP-9 can be associated with protective immunity, as largely observed with IgG antibodies against the *P. falciparum* MSP-3 protein [49], [50], [51], [52], then the higher antibody levels observed in HLA-DRB1*04 and HLA-DQB1*03 carriers could be the result of several centuries of *P. vivax* selective pressure on populations from the Amazon region (Amerindians). Similarly, we also observe negative associations with HLA-DQB1*06 and the IgG response against PvMSP3_CT_ and DRB1*01 and the repetitive regions of PvMSP-9, alleles that are present in high frequency in Caucasian populations [47] that historically had less selective pressure caused by *P. vivax*. Despite the complex evolution of the HLA system and the difficulty posed to disentangle the effects of molecular mechanisms such as selection, gene conversion and recombination, we could not exclude the strong potential influence of demographic factors and past human migrations on the observed polymorphism. Amerindian, Oceanian and Taiwanese aboriginal populations usually exhibit few alleles at high frequencies and a small number of less frequent ones, probably as a consequence of rapid genetic drift [53]. Indeed, the HLA alleles found in our studied population (Amerindians) does not represent all possible haplotypes and the association found here could reflect the linkage disequilibrium in the HLA allele. In addition other unidentified risk variants are also likely to be present and studies in a larger population from different malaria endemic regions of Southeast Asia and Africa are necessary to give support to the HLA-DRB1*04 allele association with antibody responses.

We also have in mind that PvMSP-3α is highly polymorphic and recently Ribeiro et al. [54] identify 11 haplotypes among 52 field isolates in the Brazilian Amazon and Type A was the most prevalent in four different regions (States: Amazonas, Mato Grosso, Amapá and Para). Although the recombinant proteins were derived from type A Belem strain it is possible that different haplotypes circulate in Rondonia and prevent antibody recognition of recombinants proteins. Therefore, works are in progress to evaluate the genetic background of the *P. vivax* strains circulating in the studied area. On the other hand, there is also evidences that naturally exposed individuals develop cross-reactive antibodies which recognize an increasingly broad array of *P. falciparum* isolates with increasing age or exposure [55] and in *P. vivax* AMA-1 patients harbouring polymorphic haplotypes clearly demonstrated a strain-transcending (cross reactive) antibody response against synthetic peptides [56].

In conclusion, results of this study provide valuable information concerning P. vivax vaccine candidates based on Merozoite Surface Proteins. First, recombinant proteins studied here are broadly immunogenic in a naturally exposed population. Moreover, there was no evidence of a specific HLA-DR or HLA-DQ restriction for the antibody response to the PvMSP1_19_. Contrastingly, responders to PvMSP-3α and PvMSP-9 were associated with the presence of HLA-DRB1*04 and HLA-DQB1*03 allelic groups. Interestingly, HLA alleles commonly found in South America and Southeast Asia, regions with the highest proportion of *P. vivax* malaria, were associated with high frequency of antibody responses to five out of nine proteins tested. These features could increase the success rate of future clinical trials based on these vaccine candidates.

## Materials and Methods

### Study Area and Volunteers

A cross-sectional cohort study was conducted involving 276 individuals (11 to 89 years of age) from communities in the malaria endemic region of Rondônia state, Brazil, where *P. vivax* malaria accounts for more than 70% of all malaria cases in the last five years [57]. The individuals in the study population have been described elsewhere [16], [58]. Briefly, they consist of rain forest natives as well as migrants from several non-endemic areas of Brazil who have resided in the region for 10 years or more. Samples and survey data were collected in 2007, during the dry months of June-August, coinciding with the period of increased malaria transmission in Rondonia State. Samples from 24 malaria naive individuals living in non-endemic regions and who had never visited malaria transmission zones were obtained from laboratory staff volunteers (Rio de Janeiro, Brazil and Atlanta, USA) and used as controls. Written informed consent was obtained from all adult donors or from parents of donors in the case of minors. The study was reviewed and approved by the Fundação Oswaldo Cruz Ethical Committee and the National Ethical Committee of Brazil.

### Epidemiological Survey

To evaluate epidemiological factors that may influence the immune response against the recombinant proteins, all donors were interviewed upon informed consent. The survey included questions related to demographics, time of residence in the endemic area, personal and family histories of malaria, use of malaria prophylaxis, presence of malaria symptoms, and personal knowledge of malaria. Survey data was entered into a database created with Epi Info 2007 (Centers for Disease Control and Prevention, Atlanta, GA).

### Collection of Human Blood Samples and Malaria Diagnosis

Venous peripheral blood (20 ml) was collected into EDTA tubes, and peripheral blood mononuclear cells (PBMC) were isolated by Ficoll/Hypaque (Pharmacia, Piscataway, NJ) density gradient centrifugation. Plasma was stored at –20°C and thin and thick blood smears of all donors were examined for malaria parasites at 1000× magnification under oil-immersion, all slides were examined by two researchers with expertise in malaria diagnosis. Donors positive for *P. vivax* and/or *P. falciparum* at the time of blood collection were subsequently treated per the chemotherapeutic regimen recommended by the Brazilian Ministry of Health.

### HLA Genotyping of PBMC

Genomic DNA was isolated from whole blood drawn in EDTA by using a mixture of 5 ml buffer G2 (QIAamp DNA Blood Midi Kit; Qiagen Inc., Chatsworth, CA, USA) and 95 µl proteinase K (20 mg/ml). After incubation at 50°C for 1 h the DNA was ethanol precipitated, collected with a glass stick and transferred into distilled water. DNA concentration and quality was checked with a NanoDrop ND-1000 spectrometer (Thermo Fisher Scientific Inc., Waltham, MA, USA). Sequence-specific oligonucleotide probes (SSOPs) were used by Luminex Xmap technology in order to determine the HLA class II allelic groups of studied individuals. Briefly, the system is based on probe arrays bound to color-coded plastic microspheres, and locus-specific biotinylated primers for HLA-DRB1 and HLA-DQB1 loci (LABType, One Lambda Inc, Canoga Park, CA, USA). Biotinylated amplicons were denatured to ssDNA and incubated with DNA complementary probes immobilized on fluorescent coded microspheres (beads) followed by incubation with R-Phycoerythrin conjugated to streptavidin. After hybridization, the samples were analyzed with Luminex Flow Analysis equipment. The HLA Visual 2.0 software (One Lambda, CA) analysis program deduces the HLA-DRB1 and HLA-DQB1 allelic groups. High resolution PCR-SSP typing was also used with the same method to define the DRB1*04 alleles, as these loci have been associated with responders to malaria antigens.

### Recombinant Proteins

The expression and purification of the recombinants proteins were performed as previously described [16], [58], [59]. Nine recombinant proteins derived from *P. vivax* (Belem strain) were produced. These include PvMSP-1 sequence representing the 19 kDa C-terminal fragment (MSP1_19_), PvMSP-3α sequence representing the near full length protein (MSP3_FL_), the N-terminal region (MSP3_NT_), the first block of repeats (MSP3_RI_), the second block of repeats (MSP3R_II_), and the C-terminal region (MSP3_CT_), and PvMSP-9 sequence representing the N-terminal domain (MSP9_NT_), the second block of tandem repeats (MSP9R_1I_) and the first and second block of tandem repeats MSP9_RI-RII._

### Antibody Assays

Plasma samples from study participants were screened for the presence of naturally acquired antibodies against the nine recombinant proteins: PvMSP-3α (MSP3_FL_, MSP3_RI_, MSP3_RII_, MSP3_CT_, MSP3_NT);_ PvMSP-9 (MSP9_RIRII_, MSP9_RII_, MSP9_NT_) and PvMSP-1 (PvMSP1_19_) by ELISA. Briefly, maxisorp 96-well plates (Nunc, Rochester, NY) were coated with PBS containing 2 µg per ml of each recombinant protein. After overnight incubation at 4°C the plates were washed with PBS containing 0.05% Tween 20 (PBS-Tween) and blocked with PBS-Tween containing 5% non-fat dry milk (PBS-Tween-M) for 2 h at 37°C. Individual plasma samples diluted 1∶100 PBS-Tween-M were added in duplicate wells and the plates incubated at room temperature for 1 h. After four washes with PBS-Tween, bound antibodies were detected with peroxidase-conjugated goat anti-human IgG (Sigma, St Louis) followed by o-Phenylenediamine and hydrogen peroxide. The absorbance was read at 492 nm using an ELISA reader (Spectramax 250, Molecular Devices, Sunnyvale, CA). The results for total IgG were expressed as Reactivity Indexes (RI), which were calculated by dividing the mean optical density of tested samples by the mean optical density plus 3 standard deviations of 24 non-exposed control individuals living in non-endemic areas of malaria (OD values higher than 0.089 to PvMSP1; 0,094 to PvMSP-9RIRII; 0,091 to PvMSP9RII; 0,074 to PvMSP9NT; 0,101 to PvMSP3FL; 0,081 to PvMSP3BLI; 0,092 to PvMSP3CT; 0,091 to PvMSP3NT). As positive controls we used five plasmas from exposed native individuals that presented high OD levels for all three proteins. Subjects were scored as positive to the corresponding antigen if the RI was higher than 1.

### Statistical Analysis

Survey data was recorded and entered into a database created with Epi Info 2007 (Centers for Disease Control and Prevention, Atlanta, GA). Analyses were done using Predictive Analytics Software, PASW (version 17.0; SPSS Inc., Chicago, IL, USA) and Prism version 5 (GraphPad Software Inc., San Diego, CA). Differences in medians of past malaria infections and time since the last infection for the study population data were tested by non-parametric Mann-Whitney. Student’s t test was used to compare means of normally distributed data of reactivity indexes of IgG antibodies. Differences in gender proportions were evaluated by chi-square (Χ^2^) test. Allelic groups were grouped by DR status and data were descriptively summarized using frequencies and percentages for all categorical variables. Overall associations of immunologic responses with the alleles from each HLA-DRB1* and HLA-DQB1* loci were evaluated by comparing the allele frequencies between seronegative subjects and seropositive subjects using standard contingency tables. For a locus with K alleles, a 2×K table was constructed by computing allele counts for each of the two groups. Each person contributed two observations to the table (one for each allele). Rare alleles, defined as those with less than five occurrences among subjects, were all pooled into a category labeled “other" for analysis. To evaluate global differences in allele distribution, we performed analyses using simulation methods as implemented in the software PASW. This approach randomly generates new cell counts for contingency tables under the null hypothesis of no association, while keeping the margins of the table fixed. We used an approach that compares each allele versus all others combined, resulting in multiple 2×2 tables, and used the maximum Chi-square statistic from this series of tables as a global test statistic (bipartition). All global tests assume that the two alleles from each subject are independent. Following the global tests of association, we examined individual allele effects on seropositivity using unconditional logistic regression analysis. Regression variables were created for each allele and coded as 0, 1, or 2, according to the number of copies a subject carries. Each allele variable was first included in a separate univariate logistic regression analysis to determine its association with seronegativity, ignoring the effects of other alleles. Next, to verify the influence of haplotypes, multivariate analyses were run that examined the simultaneous effects of multiple alleles at a given locus with seronegativity. Separate models were fit for each of the five loci. The low frequency of many alleles, coupled with data dependency issues, made it impossible to fit all allele count variables from one locus in a single logistic model. Because of this, we used a forward stepwise regression method to choose alleles most associated with antibody response. The significance level for entering an allele variable in the model was 0.05. Variables not included in the final stepwise model were, by default, pooled into a group against which the significant allele variables were compared. To examine the possibility of multiple effects on antibody levels, multivariate regression analysis was also performed by Generalized Linear Models (GLM). The reactivity index of IgG antibodies values were used as dependent continuous variable, HLA alleles as independent variable and time (years) of residence in endemic area (TREA), time (months) since the last malaria episode (TLI) and past malaria infections (PMI) individually as independent covariates. Interaction terms were also included (TREA+HLA; TREA+TLI; TREA+PMI) to test whether the associations between the reactivity index and HLA-DRB1* or HLA-DQB1* were significant. Partial η^2^ (*Partial eta-squared*) were also used to evaluate the power of association of each variable individually in the model. All variables included in the final locus-specific stepwise models were placed into one overall stepwise model. As before, the significance level for entering a variable in the model was 0.05. All statistical tests were two sided and, unless otherwise specified, HLA analyses were conducted using the PASW software system.
